# A New Graphic Type Differentiation of Cell Account Determination for Distinguishing Acute Periprosthetic Joint Infection from Hemarthrosis

**DOI:** 10.3390/antibiotics11101284

**Published:** 2022-09-21

**Authors:** Bernd Fink, Marius Hoyka, Elke Weissbarth, Philipp Schuster, Irina Berger

**Affiliations:** 1 Department for Joint Replacement, Rheumatoid and General Orthopaedics, Orthopaedic Clinic Markgröningen, Kurt-Lindemann-Weg 10, 71706 Markgröningen, Germany; 2 Orthopaedic Department, University Hospital Hamburg-Eppendorf, Martinistrasse 52, 20251 Hamburg, Germany; 3 Department of Orthopedics and Traumatology, Clinic Nuremberg, Paracelsus Medical University, Nuremberg, Breslauer Straße 201, 90471 Nürnberg, Germany; 4 Institute of Pathology, Klinikum Kassel, Mönchebergstraße 41-43, 34125 Kassel, Germany

**Keywords:** periprosthetic joint infection, diagnosis, leukocyte, cell count, CRP

## Abstract

Aims: This study evaluates the value of a new graphic representation of cell count data of synovial fluid in the diagnosis of acute periprosthetic joint infection (PJI). Methods: A total of 75 patients with revisions of 48 primary total knee and 27 hip arthroplasties within the first six weeks after surgery were analyzed with cultivation of the synovial fluid and determination of its cell count as well as microbiological and histological analyses of the periprosthetic tissue obtained during the revision surgery using the ICM classification. The synovial fluid was additionally analyzed for graphic representation of the measured cells using LMNE-matrices. Results: A total of 38 patients (50.7%) had an infection. The following types of LMNE matrices could be differentiated: the indeterminate type (IV) in 14.7%, the infection type (II) in 5.3%, the hematoma type (V) in 33.3%, and the mixed type (VI; infection and hematoma) in 46.7%. Differentiation of LMNE types into infection (types II and VI) and non-infection (types IV and V) resulted in a sensitivity of 100%, a specificity of 97.3%, and a positive likelihood ratio of 37.0. The cell count measurement showed a sensitivity of 78.9%, a specificity of 89.2%, and a positive likelihood ratio of 7.3 at a cut-off of 10,000 cells. The percentage of polymorphonuclear leukocytes showed a sensitivity of 34.2%, a specificity of 100%, and a positive likelihood ratio of >200 at a cut-off of 90%. Conclusion: The graphic representation of the cell count analysis of synovial aspirates is a new and helpful method for differentiating between genuine early periprosthetic infections and postoperative hemarthrosis.

## 1. Introduction

Acute periprosthetic joint infection (PJI) is a devastating complication of arthroplasty procedures with an incidence for total hip and knee arthroplasties ranging between 0.16% and 0.6% [1,2]. The diagnosis of early PJI is often difficult as the normal postoperative peri-incisional swelling and erythema make it difficult to distinguish an early postoperative infection from the normal postoperative course [1,2,3]. Moreover, recurrent postoperative hemarthrosis after total joint arthroplasty has an incidence between 0.3% and 1.6% and can mimic the clinical symptoms of an early infection [4,5].

An important diagnostic method for PJI is the determination of the leukocyte count (WBC) in the joint synovial fluid. Some authors consider it to be the most important diagnostic parameter [1,2,3,6], and it is certainly one key parameter in the MSIS criteria and the more recent ICM criteria [7,8,9]. The cut-off value for the leukocyte number given in the study of Bedair et al. [1], above which the finding is to be assessed as positive for an acute PJI with a high sensitivity, is 10,700 cells/μL. Similarly, Kim et al. [2] reported 11,200 cells/μL as a cut-off value in their study of total knee arthroplasties, Yi et al. [3] reported 12,800 cells/μL for total hip arthroplasties, and Yu et al. [6] and Sukhonthamarn et al. [10] reported 8910 cells/μL and 10,170 cells/μL, respectively, for total hip and knee arthroplasties. The International Consensus Group meeting set the cutoff value at 10,000 cells/µL for the detection of acute periprosthetic infection [11]. However, these cut-off values correspond to leukocyte values that may also be present for normal blood, so postoperative hematomas in the joint may also exhibit leukocyte values in this range [1,3].

Another important parameter reported is the percentage of polymorphonuclear leukocytes (PMN) in the aspirate [7,8,9,11], and the cut-off value was determined to be 89% in the studies by Bedair et al. [1] and Yi et al. [3] and at a similar level in the study by Kim et al. [2] but was 79.5% in Sukhonthamarn et al. [10]. The International Consensus Group meeting set the cut-off value to 90% PMN in the aspirate [11]. However, the percentage of polymorphonuclear leukocytes in the aspirate can also be influenced by the presence of a hematoma.

Additionally, the serum CRP level, which was agreed by the consensus meeting to have a threshold value of 100 mg/L for an acute periprosthetic infection, may attain this level in an early postoperative period in the absence of an infection [1,2,3,11]. Therefore, differentiation of an acute periprosthetic infection from a postoperative hematoma without infection appears to be challenging.

**Figure 1:**
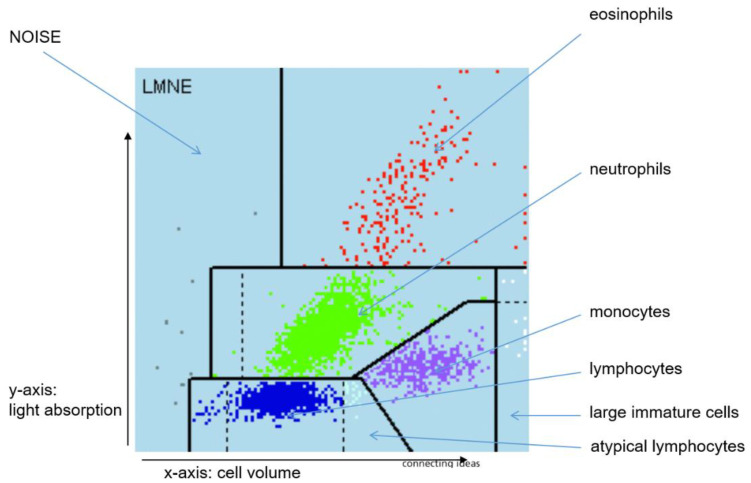
LMNE matrix with the different fields for the leukocyte populations and the NOISE area.

In a study by Fink et al. [12], the authors demonstrated that graphical representation of the cell count analysis in the aspirate represents a new and helpful tool in the diagnosis of late periprosthetic infection [12]. Here, the so-called LMNE matrix differentiates the five cell types—eosinophils, neutrophils, monocytes, lymphocytes, and basophils as well as atypical lymphocytes and large, immature cells—depending on their cell volume (*x*-axis) and their light scattering or refraction and absorption (*y*-axis) (Figure 1). Thus, different patterns in the LMNE matrix might be expected for a postoperative hematoma, a postoperative acute periprosthetic infection, and possibly a mixed type. Therefore, the aim of the present study was to answer the following questions or to test the hypotheses:

1. Can different types (infection type, hematoma type, and mixed type of infection and hematoma) be differentiated in the LMNE matrix?
2. Does this type differentiation help in the diagnosis of acute periprosthetic infection?

## 2. Materials and Methods

This retrospective analysis of prospectively collected data in the data base included 101 patients (54 women, 47 men) who had undergone revision surgery within the first 6 weeks after primary implantation (59 total knee replacements, 42 total hip replacements) between 2014 and 2021. They all underwent a prior aspiration of the joint. Patients with systemic inflammatory diseases, such as rheumatoid arthritis, were excluded because these diseases can demonstrate leukocytes in the joint irrespective of the presence of a hematoma or a PJI [13]. Moreover, 11 patients who were treated with antibiotics prior the aspiration were excluded because antibiotics reduce the count of white cells in the synovia as well as the CRP-level in the serum [14]. The remaining 75 patients (39 women, 36 men) had revisions of 48 total knee replacements and 27 hip replacements. The mean age of the patients was 71.6 ± 9.7 years (51–93 years). Revision surgery was performed 25.9 ± 11.9 days (7–44 days) after the primary implantation. The cause of revision surgery was a suspected early infection or an infected hematoma in 63 cases and revision of the hip joint because of instability with dislocation in 12 cases. The joint aspiration was performed 24.8 ± 10.8 days (7–43 days) after the primary implantation. The joint aspiration techniques were carried out under sterile conditions.

The cell count in the aspirate was determined as follows: At least 1 mL synovial fluid was filled in an EDTA tube for determining the cell count with the laboratory diagnostic device ABX Pentra XL 80 (Horiba Medical, Montpellier, France). The ABX Pentra XL 80 is a laboratory diagnostic device for analysis of the cell count and the WBC-differentiation of blood and other bodily fluids. Here the so-called 5-DIFF mode was selected from the various processing modes available. A total of 26 laboratory parameters were recorded, including the five cell types—eosinophils, neutrophils, monocytes, lymphocytes, and basophils as well as atypical lymphocytes and large, immature cells. These cell types were graphically mapped in a so-called LMNE matrix depending on their cell volume (*x*-axis) and their light scattering or refraction and absorption (*y*-axis) (Figure 1). The analysis is based on an impedance measurement, flow cytometry, and cytochemistry. The volume difference of the cells due to the impedance measurement is shown graphically on the *x*-axis and the differentiation of the light absorption in flow cytometry on the *y*-axis of the LMNE matrix. This enables the graphical assignment and thus differentiation of the four leukocyte populations: lymphocytes, monocytes, neutrophils, and eosinophils (Figure 1). The so-called NOISE area of the LMNE matrix contains impurities. This graphical assignment of leukocyte population in the LMNE matrix is an established method in hematology [15,16,17].

**Figure 2:**
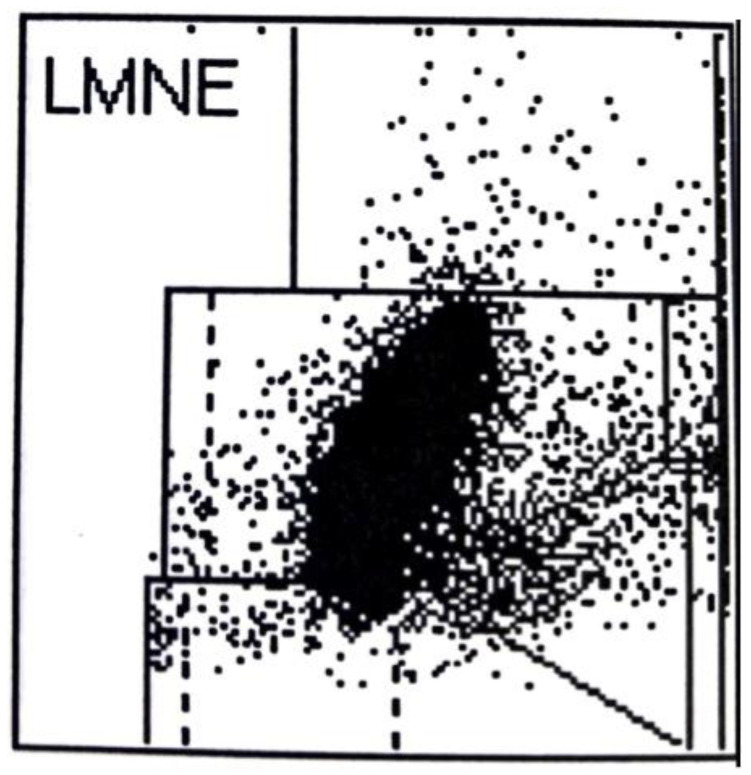
LMNE matrix of a type II (infection type) with a cluster, or increase, in the area of the neutrophils of a 68 year old male patient 5 weeks after implantation of a knee arthroplasty. The measured cell count was 21,900 cells/µL with 89.7% PMN and a CRP of 27.1 mg/L.

**Figure 3:**
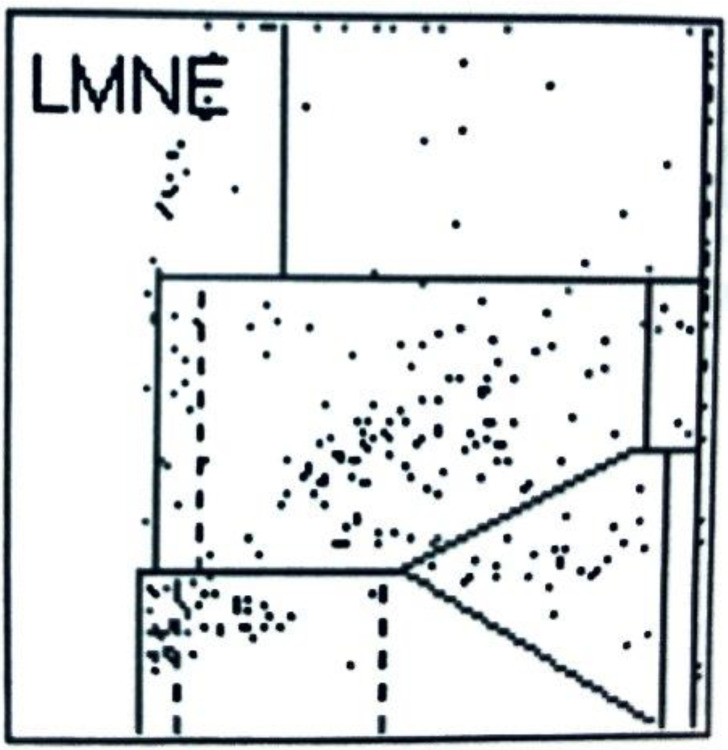
LMNE matrix of a type IV (indeterminate type) with a cluster in the area of the lymphocytes and a small cluster in the are of the neutrophils of a 87 year old female patient with an aspirate of the hip arthroplasty 4 weeks after surgery, before dislocation. The measured cell count was 300 cells/µL with 51.2% PMN and the CRP was 4 mg/L (<5 mg/L is normal).

**Figure 4:**
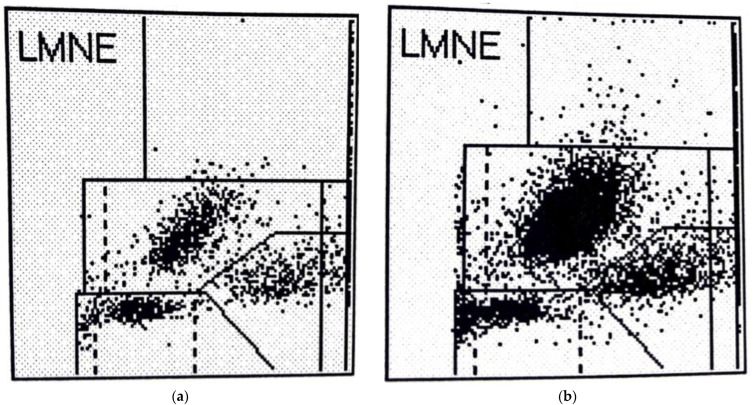
(**a**): LMNE matrix of a type V (hematoma type) of a 71 year old woman 6 weeks after hip arthroplasty with a hematoma. The measured cell count was 3560 cells/µL with 63.5% PMN; the CRP was 143.7 mg/L (<5 mg/L is normal). (**b**): LMNE matrix of a type V (hematoma) 74-year-old female patient with a hematoma 6 weeks after hip arthroplasty. There is a cluster in the field of the lymphocytes and another clear cluster in the field of the neutrophils. The measured cell count was 11,400 cells/µL with 79.1% PMN and the CRP was 86.9 mg/L.

**Figure 5:**
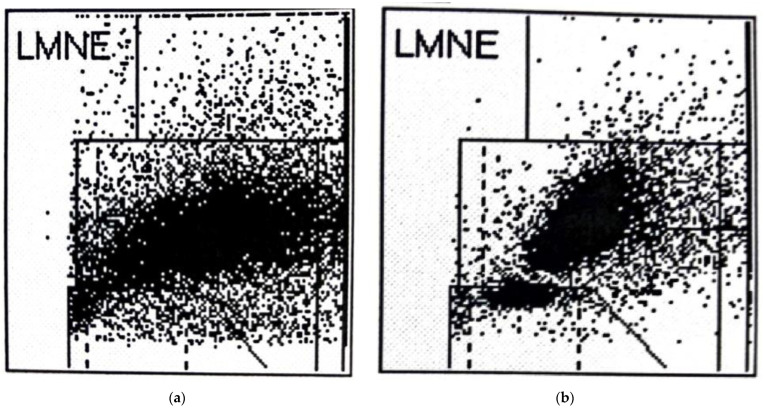
(**a**): LMNE matrix of a type VI (combined type II of hematoma and infection) 3 weeks after implantation of a hip arthroplasty with a cluster in the area of the neutrophil leukocytes and increases in the other areas of the white blood cells in a 57-year-old male patient with an early periprosthetic joint infection. The measured cell count was 62,750 cells/µL with 83.8% PMN and a CRP of 255.1 mg/L. (**b**): LMNE matrix of a type VI (combined type II of hematoma and infection) 3 weeks after implantation of a hip arthroplasty with a cluster in the area of the neutrophil leukocytes and increases in the other areas of the white blood cells in a 53-year-old male patient with an early periprosthetic joint infection. The measured cell count was 32,750 cells/µL with 80.4% PMN and the CRP was 66.9 mg/L.

In addition to the four LMNE types already defined in the study of late periprosthetic infections by Fink et al. [12] (type I = abrasion type, type II = infection type with a cluster in the neutrophil field without appreciable clusters in the other leukocyte fields (Figure 2), type III = mixed type of abrasion and periprosthetic infection, type IV = indeterminate type (Figure 3)), two further types were defined: type V as the hematoma type (with smaller clusters in the fields associated with eosinophils, monocytes, lymphocytes and/or neutrophils) (Figure 4a,b) and type VI as a mixed type II between infection (with large clusters in the neutrophil field) and hematoma (with the corresponding smaller clusters for the other white blood cells) (Figure 5a,b).

The evaluation and assignment of the matrices to these different types were performed twice by 2 independent examiners (BF and MH) and without the knowledge of the histology and other results. This showed a high level of reliability, with inter-rater and intra-rater reliability coefficients of 0.98 and of 0.98, respectively.

Additionally, the harvested fluid was immediately introduced into pediatric blood culture bottles containing BD BACTEC-PEDS-PLUS/F-Medium (Becton Dickinson, Heidelberg, Germany) and incubated for 14 days [18]. Serum CRP-level (mg/L) was measured by a particle-enhanced turbidimetric immunoassay (Cobas C303; Roche, Basel, Switzerland) in all cases.

Within the revision surgery itself, samples were taken from five different areas close to the prosthesis (synovium and periprosthetic tissue) for aerobic and anaerobic cultures. In addition, five samples from the synovium and the periprosthetic connective tissue membrane were obtained for histological assessment. Perioperative antibiotics were only administered after all the samples had been taken. The biopsy samples were each placed in sterile tubes and transferred together with the aspirated fluid to the microbiological laboratory within an hour of sampling. Patient specimens were processed immediately after arrival at the laboratory. PEDS culture vials were treated with Fastidious Organism Supplement (FOS) (Becton Dickinson, Heidelberg, Germany), and incubated using the BD BACTEC 9050 automatic blood culture system (Becton Dickinson, Heidelberg, Germany). Turbid broths were subcultured onto appropriate agar plates. Microorganisms were identified by standard microbiological procedures including biochemical characterization with the API system (BioMerieux, Nuertingen, Germany) in case of anaerobic strains or anaerobic bacterials. Antibiotic susceptibility testing was performed by disk diffusion or dilution methods, according to the Clinical and Laboratory Standards Institute (CLSI) guidelines. In all other cases we used Vitek II (BioMerieux, Nuertingen, Germany) for indentification and antibiotic susceptibility testing. All the samples were incubated for 14 days [18]. The results together with results of the aspiration were analyzed according to the ICM-criteria 2018 [7,8,9,11]. Hereby, the results were rated as periprosthetic joint infection (PJI) when the sum of the diagnostic results was at least 6. For the histological analysis of the periprosthetic tissue, in addition to counting the polymorphonuclear leukocytes per high power field, the classification by Krenn et al. [19,20] and Morawietz et al. [21] was used, which differentiates between abrasion type (I), infection type (II), mixed type (III), and indeterminate type (IV).

Statistical evaluation was performed using SPSS for Windows (version 22, IBM Corp.; Armonk, NY, USA). The chi-square test was used for comparison of nominal variables, the Mann–Whitney U test was used for evaluation of non-parametric data. All reported *p*-values are two-tailed, with an alpha level <0.05 considered significant. For evaluation of test quality sensitivity and specificity, positive and negative predictive value and accuracy as well as positive and negative likelihood ratios and accuracy were calculated. A receiver-operating characteristic curve (ROC curve) was calculated for any continuous parameter, with the area under the curve being defined as follows: 0.6–0.7 poor, 0.7–0.8 fair, 0.8–0.9 good, and 0.9–1.0 excellent. Unless otherwise stated data are presented as mean ± standard deviation (and range) or median and (interquartile range, 25–75%).

## 3. Results

A total of 38 patients (50.7%) had an infection according to the ICM criteria. The distribution of the detected microorganism is shown in Table 1. The nucleated cell count in the aspirate from patients with infections was 17,400 (12,100–32,870) cells/µL, significantly higher than that in the patients without infection, which was 1600 (300–4300) cells/µL (*p* < 0.001). However, four patients without infection had a higher cell count and eight patients with infection had a lower cell count than the threshold of 10,000 cells/µL set by ICM [7,8,9,11] (Table 2). The percentage of polymorphonuclear leukocytes was also significantly higher in patients with infections, 86.0% (79.6–90.9%), than in patients without infection, 61.5% (50.4–72.8%) (*p* < 0.001). Depending on the cutoff value selected, the resulting distributions and precision were as shown in Table 2. The CRP level was also significantly higher in the patients with infection, 96.9 (49.1–198.7) mg/L, compared with 35.1 (9.8–82.2) mg/L in patients without infection (*p* < 0.001). According to the selected cut-off values, the resulting distributions and precision were as shown in Table 2.

**Figure 6:**
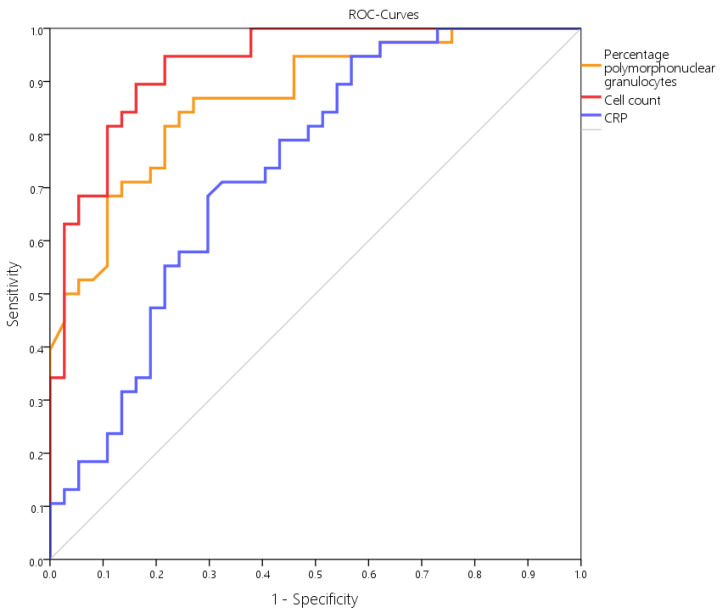
ROC curves of the parameters cell count, percentage PMN, and CRP. Area under the curve (AUC) was 0.932 for cell count, 0.868 for %PMN, and 0.734 for CRP.

When comparing these three parameters, only the cell count showed a higher accuracy and likelihood ratio (Table 2). Further, when calculating the area under the curve (AUC) in the receiver operation curve (ROC), the cell number showed a higher AUC of 0.932 than the percentage of PMN (AUC = 0.868) and serum CRP value (AUC = 0.734) (Figure 6). The combination of cell count and percentage of polymorphonuclear leukocytes resulted in improved specificity but lower sensitivity (Table 2).

When LMNE matrices were evaluated, type IV (indeterminate type) (Figure 3) was present in 11 cases and type V (hematoma type) (Figure 4a,b) in 25 cases, which were scored as types without infection. Type II (infection type) (Figure 2) was identified 4 times, and type VI (mixed type of infection with hematoma) (Figure 5a,b) was identified 35 times, resulting in 39 cases scored as infection in the LMNE analysis (Table 2). Overall, the LMNE matrix evaluation showed a very high and significantly better accuracy of 98.7%, a sensitivity of 100%, a specificity of 97.3%, a positive predictive value of 97.4%, a negative predictive value of 100%, a positive likelihood ratio of 37.0, and a negative likelihood ratio of 0.0. A non-infected hematoma was misclassified as an infected LMNE type VI (mixed type of infection with hematoma) one time (Table 2). The cultivation of the aspirate showed high sensitivity (84.2%) and specificity (100%) but lower sensitivity than the LMNE matrix evaluation (Table 2).

## 4. Discussion

The chosen type classification of LMNE matrices with a differentiation between types with infection (type II (infection type) and type VI (infection + hematoma type) and types without infection (type IV (indeterminate type) and type V (hematoma type) had a high sensitivity of 100% and a specificity of 97.3%. Therefore, this type classification could reliably distinguish between cases with an acute periprosthetic infection and cases without infection in the early post-surgery phase. It was superior in accuracy to all other diagnostic parameters (pure cell count and percentage of PMN in the aspirate as well as CRP value in the serum). Therefore, this method can be used to differentiate infections from hematoma, which appears to be of particular importance since postoperative edema and swelling can make it difficult to clinically distinguish these two situations. Moreover, the cell count in a hemarthrosis can be at the level of the threshold for infection described by the ICM and other authors [1,2,3,6,7,8,9,10,11].

LMNE matrix evaluation could help to differentiate infections from hematomas especially in the cases with cell counts in the borderline range of the threshold value chosen by the ICM, because cluster plots in the fields representing lymphocytes, basophils, and/or eosinophils show that a significant part of the cluster in the neutrophil leukocyte field must be due to a hematoma and therefore cell count values in the borderline range can be corrected downward and thus not be interpreted as an infection (Figure 4b).

The other LMNE matrix types I (abrasion type) and type III (mixed type of infection and abrasion) defined in the publication of Fink et al. [12] did not occur in this study because there is no significant abrasion during the early postoperative phase and therefore abraded particles were not found.

The cultivation of the aspirate had almost as good accuracy, sensitivity, and specificity as the LMNE matrix evaluation. The disadvantage of cultivation compared to the other tests of the aspirate is that the results are only available after a few days, which means that valuable time for the treatment of an acute periprosthetic infection is lost [22].

Serum CRP demonstrated the lowest diagnostic value in the present study. On the one hand, this may be due to it usually taking 2 to 4 weeks after surgery for the CRP value to normalize. In some cases, this might take up to 60 days and thus higher CRP values may be present during this time in the absence of infection [23,24,25]. On the other hand, the actual CRP level in the serum lags approximately 60 h behind the current situation in situ, so the onset of infection in the joint might already have occurred, while the CRP value in the serum is still below the defined threshold value (Figure 2 and Figure 5b) [22,23,24].

Even though this is the first description of such a type differentiation in the analysis of cell count data for assessing early periprosthetic infections, this study has some weaknesses. One of the limitations is the retrospective analysis of prospective collected data. However, relatively rare events, such as periprosthetic joint infection of TKA and THA within the first 6 weeks after surgery (0.16% and 0.6% incidence in the reports by Bedair et al. [1] and Yi et al. [2]), are difficult to study in a prospective manner and are often better suited for retrospective analyses. Moreover, all other studies on the diagnostic value and cut-off analysis of the cell count in the aspirate in periprosthetic early infections in the literature were retrospective [1,2,3,6,10]. Furthermore, the number of cases is relatively small. This is also due to the low necessity for revisions within the first 6 weeks after implantation of a knee or hip prosthesis.

The present study represents the first pilot study on the topic of graphical representation of cell count analysis in suspected early infections. However, the number of cases was sufficient to demonstrate the positive effect of graphical presentation of aspirate cell count data for the differentiation of hematoma and infection. Nevertheless, this is still a first pilot study and the type differentiation proposed here must be verified by further studies with larger patient numbers. Furthermore, this type classification, as well as that in histopathology, is somewhat dependent on the personal interpretation and experience of the examiner. Even if the reliability of this type classification was very high in this study, it does not rule out a certain subjectivity in the interpretation of the LMNE matrices. Furthermore, such an LMNE matrix of synovial aspirate cannot be created by all cell counting devices. This is because the creation of the LMNE matrix requires measurement of the light absorption of the cells or particles and many devices for determining the cell count only measure the scattered light and the size of the cells or particles.

On the other hand, the strength of this study lies in the uniformity of the diagnostic procedure and, above all, in the verification of an infection in all cases by using the results of several diagnostic criteria applied to samples obtained before and during the revision operation (cultivation of the aspirate as well as of five tissue samples, histology, and CRP determination).

## 5. Conclusions

The graphic representation of the cell count analysis of synovial fluid is a new and helpful method for distinguishing between true early periprosthetic infections with increased leukocyte counts and high cell counts due to hemarthrosis. It is especially helpful in cases with cell counts and percentages of PMN in the cut-off range and thus in making a distinction between acute periprosthetic infection and postoperative hematoma. The value of this new graphical representation of synovial cell count analysis for the diagnosis of early periprosthetic infections should be verified in further multicenter-studies as already mentioned for this method in the diagnosis of late periprosthetic infection [12].

## Figures and Tables

**Table 1:** Distribution of the detected microorganism of the 38 patients with acute periprosthetic infection.

Microorganism	Early Infection N = 38
*Staphylococcus aureus*	18 (47.4%)
*Staphylococcus epidermidis—MSSE*	12 (31.6%)
*Staphylococcus epidermidis—MRSE*	1 (2.6%)
*Staphylococcus capitis*	3 (7.9%)
*Staphylococcus lugdunensis*	1 (2.6%)
*Cutibacterium acnes*	2 (5.3%)
*Streptococcus agalactiae*	1 (2.6%)

MSSE = Methicillin sensible *staphylococcus epidermidis*; MRSE = Methicillin resistant *staphylococcus epidermidis.*

**Table 2:** Diagnostic values for the different parameters with different cut-offs.

		Infection			Accuracy	98.7%	LR Pos.	LR Neg.
		yes	no		Sensitivity	100.0%		
LMNE	Infection	38	1	39	Specificity	97.3%	37.0	0.0
	No infection	0	36	36	PPV	97.4%		
		38	37	**75**	NPV	100.0%		
		Infection			Accuracy	62.7%	LR pos.	LR neg.
		yes	no		Sensitivity	47.4%		
CRP	≥100 mg/L	18	8	26	Specificity	78.4%	2.19	0.67
	<100 mg/L	20	29	49	PPV	69.2%		
		38	37	**75**	NPV	59.2%		
		Infection			Accuracy	64.0%	LR pos.	LR neg.
		yes	no		Sensitivity	50.0%		
CRP	≥90 mg/L	19	8	27	Specificity	78.4%	2.31	0.64
	<90 mg/L	19	29	48	PPV	70.4%		
		38	37	**75**	NPV	60.4%		
		Infection			Accuracy	65.3%	LR pos.	LR neg.
		yes	no		Sensitivity	60.5%		
CRP	≥75 mg/L	23	11	34	Specificity	70.3%	2.04	0.56
	<75 mg/L	15	26	41	PPV	67.6%		
		38	37	**75**	NPV	63.4%		
		Infection			Accuracy	84.0%	LR pos.	LR neg.
		yes	no		Sensitivity	78.9%		
Cell Count	≥10,000/µL	30	4	34	Specificity	89.2%	7.3	0.24
	<10,000/µL	8	33	41	PPV	88.2%		
		38	37	**75**	NPV	80.5%		
		Infection			Accuracy	76.0%	LR pos.	LR neg.
		yes	no		Sensitivity	73.7%		
PME [%]	≥79.5	28	8	36	Specificity	78.4%	3.41	0.34
	<79.5	10	29	39	PPV	77.8%		
		38	37	**75**	NPV	74.4%		
		Infection			Accuracy	66.7%	LR pos.	LR neg.
		yes	no		Sensitivity	34.2%		
PME [%]	≥90	13	0	13	Specificity	100.0%	>200.0	0.66
	<90	25	37	62	PPV	100.0%		
		38	37	**75**	NPV	59.7%		
		Infection			Accuracy	72.0%	LR pos.	LR neg.
		yes	no		Sensitivity	50.0%		
Cell Count ≥ 10,000/µL AND PMN ≥ 79.5%	yes	19	2	21	Specificity	94.6%	9.25	0.53
	no	19	35	54	PPV	90.5%		
		38	37	**75**	NPV	64.8%		
		Infection			Accuracy	64.0%	LR pos.	LR neg.
		yes	no		Sensitivity	28.9%		
Cell Count ≥ 10,000/µL AND PMN ≥ 90.5%	yes	11	0	11	Specificity	100.0%	>200.0	0.71
	no	27	37	64	PPV	100.0%		
		38	37	**75**	NPV	57.8%		
		Infection			Accuracy	92.0%	LR pos.	LR neg.
		yes	no		Sensitivity	84.2%		
Cultures of aspirate	positive	32	0	32	Specificity	100.0%	>200.0	0.16
	negative	6	37	43	PPV	100.0%		
		38	37	**75**	NPV	86.0%		

PMN: Polymorphonuclear leukocytes; PPV: positive predictive value; NPV: negative predictive value; LR pos: likelihood-ratio positive; LR neg: likelihood-ratio negative.

## Data Availability

The data presented in this study are available on request from the corresponding author. The data are not publicly available due to privacy.
